# The effect of statin treatment on intratumoral cholesterol levels and LDL receptor expression: a window-of-opportunity breast cancer trial

**DOI:** 10.1186/s40170-020-00231-8

**Published:** 2020-11-23

**Authors:** Maria Feldt, Julien Menard, Ann H. Rosendahl, Barbara Lettiero, Pär-Ola Bendahl, Mattias Belting, Signe Borgquist

**Affiliations:** 1 Department of Clinical Sciences Lund, Division of Oncology and Pathology, Lund University, Lund, Sweden; 2 Department of Oncology, Skåne University Hospital, Lund, Sweden; 3 Department of Immunology, Genetics and Pathology, Uppsala University, Uppsala, Sweden; 4 Department of Oncology, Aarhus University Hospital, Aarhus, Denmark

**Keywords:** Breast cancer, Statin, Cholesterol, LDL receptor

## Abstract

**Background:**

Deregulated lipid metabolism is common in cancer cells and the mevalonate pathway, which synthesizes cholesterol, is central in lipid metabolism. This study aimed to assess statin-induced changes of the intratumoral levels of cholesterol and the expression of the low-density lipoprotein receptor (LDLR) to enhance our understanding of the role of the mevalonate pathway in cancer cholesterol metabolism.

**Methods:**

This study is based on a phase II clinical trial designed as a window-of-opportunity trial including 50 breast cancer patients treated with 80 mg of atorvastatin/day for 2 weeks, between the time of diagnosis and breast surgery. Lipids were extracted from frozen tumor tissue sampled pre- and post-atorvastatin treatment. Intratumoral cholesterol levels were measured using a fluorometric quantitation assay. LDLR expression was evaluated by immunohistochemistry on formalin-fixed paraffin-embedded tumor tissue. Paired blood samples pre- and post-atorvastatin were analyzed for circulating low-density lipoprotein (LDL), high-density lipoprotein (HDL), apolipoprotein A1, and apolipoprotein B. In vitro experiments on MCF-7 breast cancer cells treated with atorvastatin were performed for comparison on the cellular level.

**Results:**

In the trial, 42 patients completed all study parts. From the paired tumor tissue samples, assessment of the cholesterol levels was achievable for 14 tumors, and for the LDLR expression in 24 tumors. Following atorvastatin treatment, the expression of LDLR was significantly increased (*P* = 0.004), while the intratumoral levels of total cholesterol remained stable. A positive association between intratumoral cholesterol levels and tumor proliferation measured by Ki-67 expression was found. In agreement with the clinical findings, results from in vitro experiments showed no significant changes of the intracellular cholesterol levels after atorvastatin treatment while increased expression of the LDLR was found, although not reaching statistical significance.

**Conclusions:**

This study shows an upregulation of LDLR and preserved intratumoral cholesterol levels in breast cancer patients treated with statins. Together with previous findings on the anti-proliferative effect of statins in breast cancer, the present data suggest a potential role for LDLR in the statin-induced regulation of breast cancer cell proliferation.

**Trial registration:**

The study has been registered at ClinicalTrials.gov (i.e., ID number: NCT00816244, NIH), December 30, 2008.

**Supplementary Information:**

The online version contains supplementary material available at 10.1186/s40170-020-00231-8.

## Introduction

**Fig. 1:**
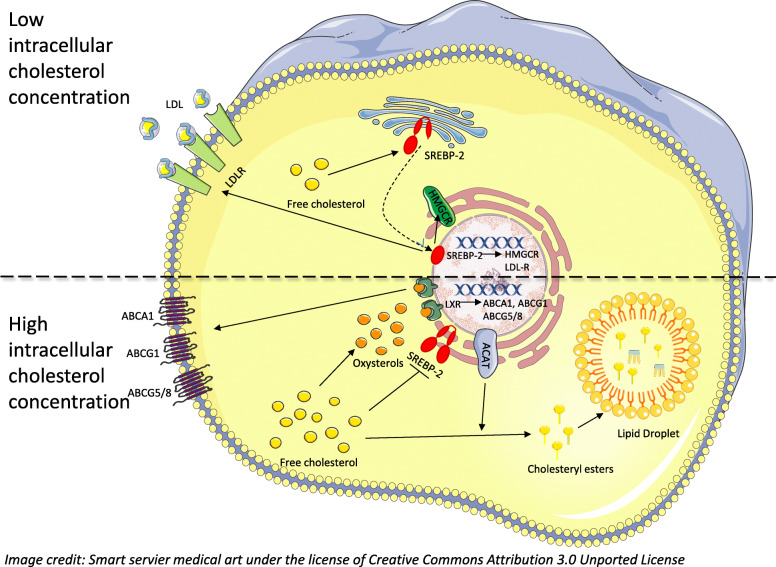
Intracellular cholesterol homeostasis. When intracellular cholesterol levels are low, SREBP-2 is delivered to the Golgi where the active, N-terminal fragment is released and translocated to the nucleus where it activates the expression of cholesterol-related genes, such as *HMGCR* and the LDL receptor. The transcriptional activation of HGMCR leads to the de novo synthesis of cholesterol via the mevalonate pathway. The activation of the transcription of the *LDLR* leads to an increase in cellular cholesterol uptake through receptor-mediated endocytosis of LDL. When cholesterol levels are high, SREBP-2 is retained in the ER. In order to prevent over-accumulation of free cholesterol in the plasma and intracellular membranes, cholesterol is converted to cholesteryl esters primarily by the enzyme ACAT. Cholesteryl esters are stored as cytosolic lipid droplets. Excess cholesterol also generates oxysterols, natural ligands for LXRs. Their binding to LXRs activates the transcription of genes involved in cholesterol efflux, including ABCA1, ABCG1, and ABCG5/8. This figure was drawn by the author M. Feldt using the image bank of Servier Medical Art. URL to the images are https://smart.servier.com/category/cellular-biology/intracellular-components. Servier Medical Art by Servier is licensed under a Creative Commons Attribution 3.0 Unported License. https://creativecommons.org/licenses/by/3.0/

Alterations in energy metabolism in cancer cells are increasingly established as a hallmark of cancer [1]. The most prominent feature of this metabolic reprogramming is the Warburg effect, which is marked by a cellular increase in glucose uptake and the use of anaerobic glycolysis in an oxygenated environment [2–4]. Accumulating evidence is now suggesting that deregulated lipid metabolism is also a common property of cancer cells, with an enhanced de novo synthesis of lipids and increased extracellular lipid recruitment as the most striking aberrations, possibly providing an advantage in cell survival due to the production of important metabolites and cell membrane remodeling [5, 6]. Altered metabolism in tumor cells is due to the activation of oncogenic signaling pathways and tumor microenvironmental stress, generating an enhanced transcription and protein synthesis of several metabolic pathway enzymes [7–11]. One important metabolic pathway within lipid metabolism is the mevalonate pathway, synthesizing cholesterol. Cholesterol is an essential component of cell membranes, modulating its fluidity and permeability, and is required for cell proliferation [12]. However, despite its critical role, aberrant levels of cholesterol can be cytotoxic, which has led to the development of complex cellular mechanisms to regulate the cellular cholesterol homeostasis, as illustrated in Fig. 1 [13]. When intracellular levels of cholesterol are low, the endoplasmic reticulum-bound sterol regulatory element-binding proteins (SREBPs) coordinate the transcriptional activation of 3-hydroxy-3-methylglutaryl-coenzyme A reductase (HMGCR), the rate-limiting enzyme of cholesterol biosynthesis, which leads to the de novo synthesis of cholesterol [14].

SREBPs also stimulate an increase in cellular cholesterol uptake, through receptor-mediated uptake of low-density lipoprotein (LDL), by activating the transcription of the LDL receptor (LDLR) [15, 16]. Newly synthesized free cholesterol can be transported to subcellular membranes by cholesterol transfer proteins, but to avoid excessive accumulation of free cholesterol, it is converted into cholesterol esters (CEs) primarily by the endoplasmic reticulum enzyme, acyl-CoA acyltransferase (ACAT) [17], and stored in intracellular lipid droplets (LDs). LDs are cytoplasmic organelles, originally thought of as static fat storage, but lately, LDs have been established as organelles with important cellular functions, including involvement in intracellular signaling and lipid homeostasis [18, 19].

The formation of LDs can either be due to excess lipid availability, a highly regulated process involving specific signaling pathways [20], or be induced by cellular stress, such as hypoxia, acidosis, inflammation, and oxidative stress [6, 21]. Excess cholesterol also generates oxysterols, i.e., natural ligands for liver X receptors (LXRs). The binding of cholesterol to LXRs triggers a conformational change in the receptor that enhances interaction with co-activator proteins, thereby facilitating the transcription of genes involved in cholesterol efflux [22]. Statins are a class of drugs exerting competitive inhibition of HMGCR, which results in the inhibition of the de novo synthesis of cholesterol in hepatocytes, leading to the upregulation of LDLR and consequently a depletion of cholesterol from plasma [15]. In recent years, attention has been drawn to the potential use of statins in cancer management [23], as their pleiotropic effects on tumor cells, such as induction of apoptosis and inhibition of angiogenesis and proliferation, have motivated their possible utility in cancer prevention and treatment [24]. In breast cancer, preclinical studies of cell lines have reported some anticancer effects by lipophilic statins [25–29]. Epidemiological data show a protective effect of statins on breast cancer recurrence and prognosis [23], and in a previous publication from the phase II trial on which this study is based, we reported a decrease in tumor proliferation following statin treatment [30]. Nevertheless, the molecular mechanisms of the anti-tumoral effects of statins are complex and remain largely undefined. The aim of this study, which is based on a translationally edged clinical trial, was to assess potential statin-induced changes in cholesterol levels and the expression of LDLR in patient tumors combined with in vitro experiments on breast cancer cells, to gain an enhanced understanding of the role of the mevalonate pathway in cancer cholesterol metabolism.

## Materials and methods

### Trial design

In this phase II window-of-opportunity breast cancer trial, all participants were prescribed an equal dose of 80 mg of the lipophilic statin, atorvastatin, for 2 weeks, during the treatment-free window between their breast cancer diagnosis and surgery. The trial was conducted at Skåne University Hospital in Lund, Sweden, as a single-center study. The trial was approved by the Ethical Committee at Lund University and the Swedish Medical Products Agency and has been registered at ClinicalTrials.gov (i.e., ID number: NCT00816244, NIH). The study adheres to the REMARK criteria [31].

### Patients

To qualify for participation in this study, patients should be diagnosed with primary invasive breast cancer, and be a candidate for radical surgery with a tumor size of 15 mm or above measured by ultrasound. Also, a performance status below 2 according to the European Cooperative Oncology Group (ECOG) and normal liver function was required for eligibility. Allergic reactions attributed to compounds with a similar biological composition to that of atorvastatin, a medical history of hemorrhagic stroke, use of cholesterol-lowering therapy (i.e., including statins, fibrates, and ezetimibe), pregnancy, or on-going hormonal replacement therapy were stated as exclusion criteria. A pre-planned number of 50 patients were enrolled in the trial, between February 2009 and March 2012. Of the 50 patients enrolled in the study, a total of 42 patients completed all study parts. No severe adverse events were reported. Further details on patient enrollment, exclusion, and inclusion criteria have been described previously [30].

### Endpoints and tumor evaluation

A statin-induced anti-proliferative tumor response measured by change in Ki-67 expression served as the primary endpoint in the clinical trial, while the purpose of this present exploratory study was to assess the impact of statins on tumor tissue cholesterol content and expression of the tumor-specific LDLR-levels. Before statin treatment initiation, patients underwent study-specific core biopsies from the breast tumor with one core biopsy being formalin-fixed immediately and one fresh frozen at −80 °C. Breast cancer surgery was performed according to standard surgical procedures, subsequent to the 2-week statin treatment, and tumor tissue was retrieved from the primary tumor storage at the Department of Pathology at Skåne University Hospital, Lund, Sweden. The serum lipid levels of total cholesterol, LDL-cholesterol, apolipoprotein B, HDL-cholesterol, and apolipoprotein A1 were measured both pre- and post-statin treatment.

### Quantification of cholesterol content

Paired tumor tissue samples obtained from the trial, before and after atorvastatin treatment, were assayed for total cholesterol levels. Quantification of tumor tissue cholesterol content was achievable in 42 of the post-atorvastatin treatment samples, and in 14 of the pre-atorvastatin treatment samples, restricted by insufficient tumor tissue in the pre-treatment core biopsies. First, lipids were extracted from the fresh frozen tumor tissue, with 200 μl of a solvent mixture of chloroform:isopropanol:igepal (7:11:0,1) (IGEPAL®CA-630, Chloroform and Isopropanol from Sigma Aldrich), sonicated using a Qsonica (model CL-19). To obtain adequate homogenization, tissue was pre-minced and sonicated for 5 min, amplitude 50%. The extracts were spun 10 min after sonication in a centrifuge at 15,000×*g*. The organic phase was then air-dried at 50 °C to remove chloroform and put under vacuum for 30 min to remove trace organic solvent. Following extraction, total cholesterol was measured using the Abcam Cholesterol Fluorometric Assay (ab65359), according to the manufacturer’s instructions. Cholesterol content was normalized to 10 mg tissue and expressed as μg cholesterol/10 mg tissue.

### Immunohistochemical evaluation of the LDLR

Formalin-fixed and paraffin-embedded tumor tissue from paired samples were cut into 3 to 4 μm sections and transferred to glass slides (Dako IHC Microscope Slides K8020), dried at room temperature, and baked in a heated chamber for 1 h at 60 °C. De-paraffinization and antigen retrieval was performed using PT Link (Dako Denmark A/S) using a high pH buffer. Immunohistochemical staining was performed in an Autostainer Plus (Dako) using a diaminobenzidine (DAB) based visualization kit (K801021-2, Dako). The primary antibody against LDLR (Abcam ab52818) was diluted 1:1000 and incubated for 30 min at room temperature. Counterstaining was performed using Mayer’s hematoxylin for 4 min. LDLR expression was evaluated by a certified senior breast pathologist (DG), and the cytoplasmic intensity was evaluated using a four-grade scale (i.e., negative, weak, moderate, or strong).

### RNA extraction of clinical samples

The Allprep DNA/RNA mini kit (QIAGEN, Valencia, CA) in a QIAcube (Qiagen) was used to extract total RNA from fresh frozen tumor samples according to the manufacturer’s instructions. The RNA integrity was assessed on an Agilent 2100 Bioanalyzer (Agilent, Santa Clara, CA) and a NanoDrop ND-1000 (NanoDrop Products, Wilmington, DE) was used to perform RNA quantification. The samples were hybridized to Human HT-12 v4.0 Expression BeadChips (Illumina Inc., San Diego, CA) in two batches at the SCIBLU Genomics Center at Lund University, Sweden (www.lth.se/sciblu). The Illumina probes were re-annotated using the R package Illumina-Humanv4.db [32]. The microarray study was conducted within another sub-study of the trial, and complete information about the comprehensive analyses of the data have been described previously [29]. Only analyses concerning the expression of the specific probes representing selected genes involved in cholesterol homeostasis are reported in this study.

### In vitro experiments

The results from the analyses of the clinical samples described above were further investigated through functional in vitro experiments, using the MCF-7 breast cancer cell line, since its estrogen receptor (ER) positive status correlates to the vast majority of the patients included in the trial.

### Cell cultures

MCF-7 cells were purchased from the American Type Culture Collection (ATCC, Rockville, MD) and were maintained at 37 °C in a humidified chamber with 5% CO_2_. Cells were cultured in Dulbecco’s Modified Eagle’s Medium (DMEM): Ham’s F-12 1:1. Media were supplemented with 10% fetal bovine serum (FBS), 2 mmol/L l-glutamine, 20 units/ml penicillin, and 20 μg/ml streptomycin. Atorvastatin calcium salt (Sigma-Aldrich) was dissolved in DMSO (Sigma) for in vitro experiments.

### Proliferation assay

MCF-7 cells were seeded in 96-well plates and treated for 72 h with 5, 10, 20, 50, and 100 μM atorvastatin to evaluate the effect of the treatment on cell proliferation using the xCELLigence Real-Time Cell Analyzer (ACEA Bioscience, Inc.).

### Quantification of cholesterol content

MCF-7 cells grown in T75 flasks and exposed to 10 μM atorvastatin at the indicated time points were assayed for total cholesterol levels, as described above. After extraction, total cholesterol was measured using the Abcam Cholesterol Colorimetric Assay (ab65359), according to the manufacturer’s instructions. Cholesterol content was normalized to 1 × 10^6^ cells and expressed as μg cholesterol/mL.

### Lipid droplet staining

To evaluate statin-induced effects on neutral lipid storage, MCF-7 cells were grown in 12-well plates (VWR) and exposed to different concentrations of atorvastatin (0-10 μM) for 24-72 h. After fixation in 3% paraformaldehyde (PFA), the cells were pre-incubated in 60% isopropanol before staining with filtered Oil Red-O working solution, obtained by mixing three parts of Oil Red-O stock (Sigma Aldrich) and two parts of deionized water for 10 min at room temperature. Excess dye was rinsed by serial washing steps in 60% isopropanol, 10% isopropanol, and PBS. Empty wells were stained in parallel and used for background subtraction. The Oil Red-O dye was extracted from the stained cells using 100% isopropanol. The absorbance reading was performed by an automatic FLUOstar OPTIMA multi-detection microplate reader (BMG Labtech) at 518 nm. The lipid content was adjusted based on the inhibitory effects of atorvastatin on cell growth rate, measured by the sulforhodamine B (SRB, Sigma) proliferation assay in parallel. Briefly, the atorvastatin-treated cells were fixed with 50 μl ice-cold 50% (w/v) trichloroacetic acid (TCA) and incubated for 1 h at 4 °C. The supernatant was discarded, and the fixed cells were stained in 50 μl SRB solution (0.4% w/v SRB in 1% acetic acid) for 20 min at room temperature. After discarding the supernatant and rinsing the plates with 1% acetic acid, the dye was dissolved in 150 μl 10 nM Tris base, and absorbance was measured at 570 nm. All experimental conditions were run in triplicate.

### Western blot analysis of the LDLR

MCF-7 cells grown in T-25 flasks were exposed to 10 μM atorvastatin for 48 h. After treatment, cell metabolism was stopped on ice and cells were washed with cold PBS, followed by lysis in cold lysis buffer containing 10 mM Tris (pH 7.6), 50 mM NaCl, 5 mM EDTA, 30 mM sodium pyrophosphate, 50 mM sodium fluoride, 100 μM sodium orthovanadate, 1% Triton X-100, 1:100 protease inhibitor cocktail (Sigma-Aldrich), 1:100 phosphatase inhibitor cocktail 2 (Sigma-Aldrich), and stored at −20 °C overnight, to enhance the lysis efficacy. Subsequently, protein content was measured by the BCA protein assay kit (Pierce). Lysates were dissolved in Laemmli buffer, boiled for 5 min and protein separation on precast 10% NuPAGE Bis-Tris gel (Novex, Life Technology) was performed. Proteins were then transferred to nitrocellulose membranes (Amersham Protran, GE Healthcare) blocked in 5% milk TBS-T and probed overnight (4 °C) with anti-LDLR (0,5 μg/ml, PA5-22976, Thermo Fisher Scientific, IL, USA) and anti-GAPDH (1:1000, MAB374, Merck-Millipore, Darmstadt, Germany) antibodies in 5% w/v BSA-TBST. After incubation with the primary antibodies, the membranes were washed three times with 5% skimmed dry milk in TBS-T and incubated with the horseradish peroxidase (HRP)-conjugated secondary antibodies (Sigma-Aldrich) for 1 h at 4 °C. Thereafter, the membranes were washed and immunoreactive bands were developed for 5 min using enhanced chemiluminescent reagents (Super Signal West Dura, Extended duration substrate, Thermo Scientific). Then the membranes were removed from the chemiluminescent solution, wrapped in plastic sheet protectors, and the signal captured by auto exposure to a CCD (Alpha Innotech Fluorchem FC2). Later the signal intensities for specific bands on the Western blots were semi-quantified by densitometry using the 1-D analysis software (AlphaView v 3.0.3.0 ProteinSimple, San Jose, Cal., USA).

### Statistical analysis

Regarding the clinical samples, changes in intratumoral cholesterol levels, LDLR protein expression, and gene expression of the cholesterol homeostasis genes between pre- and post-atorvastatin treatment samples were evaluated using the Wilcoxon matched-pairs signed-rank test. For comparison between the normal and the cholesterol-rich samples, categorical variables were compared between the grouped samples using Pearson’s chi-square test and ordinal variables were compared between groups with the Mann-Whitney *U* test. Spearman’s rho was used as a measure of the correlation between intratumoral cholesterol levels and Ki-67, and between the upregulation of LDLR and Ki-67. All tests were two-sided, and *P* values were interpreted as a measure of the level of evidence against a null hypothesis, as suggested by Benjamin et al. [33], i.e., suggestive evidence for *P* values in the range from 0.005 to 0.05 and significant evidence below 0.005. For the in vitro experiments, changes in cholesterol and lipid droplet content were evaluated using a two-way ANOVA. The results of the cholesterol levels are expressed as the mean ± standard deviation of three separate experiments and of the lipid droplet content as the geometric mean ± 95% confidence interval of the geometric mean of three separate experiments. Regarding the Western blot analysis of the LDLR, results are expressed as the geometric mean ± 95% confidence interval of the geometric mean of three separate experiments. Pairwise comparisons of geometric means were done with Student’s *t* test. The software package IBM SPSS Statistics Version 22, GraphPad Prism 8.3.0, and Stata version 16.0, StataCorp LLC, were used for the data analysis.

## Results

### Patient characteristics, tumor data, and serum lipids

The average age among all 42 patients was 63 years (range, 35–89 years) at the time of inclusion. All 42 tumors were invasive breast cancers, and the average pathological tumor size was 21 mm, ranging from 6 to 33 mm. Most tumors were Luminal A-like breast cancer, and 79% were histological grade 2 or 3. As previously reported [34], the mean decrease of serum total cholesterol following statin treatment was 64%, with a 47% decrease in LDL cholesterol. Correspondingly, there was a 61% decrease in apolipoprotein B. Both HDL cholesterol [34] and apolipoprotein A1 remained as expected at approximately the same levels following atorvastatin treatment.

### Analysis of tissue cholesterol content in clinical samples

**Fig. 2:**
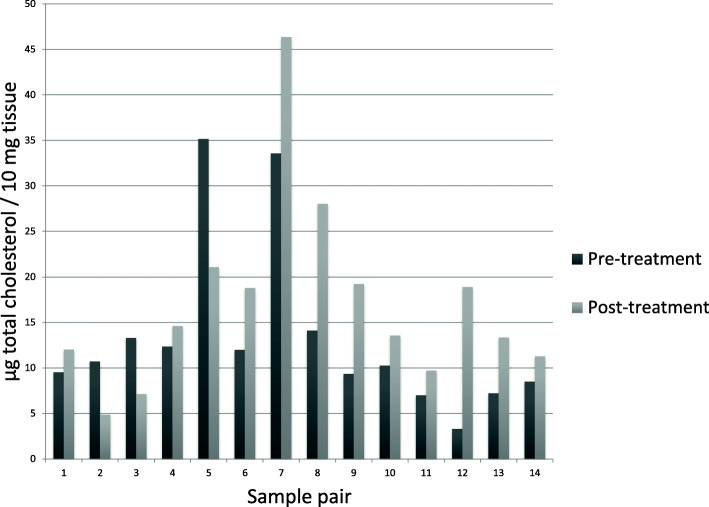
Paired samples total cholesterol levels. Total cholesterol levels in tumor tissue were measured using a cholesterol assay before and after 2 weeks of treatment with 80 mg atorvastatin daily. The tumor tissue total cholesterol content was higher in 11 of the 14 paired samples after 2 weeks of atorvastatin treatment lower than before treatment in the remaining three cases. No statistically significant differences in the levels of total cholesterol were observed [*P* = 0.11 (Wilcoxon signed-rank test)]

Analyses of the total cholesterol content in tumor tissue from the clinical samples were performed on the 14 paired tumor samples with a sufficient amount of frozen tissue. Prior to atorvastatin treatment, the total cholesterol level ranged between 3.31 and 35.15 μg total cholesterol/10 mg tissue, with a median of 10.49 μg. After atorvastatin treatment, the total cholesterol levels were ranging between 4.87 and 46.35 μg total cholesterol/10 mg tissue, with a median of 14.1. In 11 out of the 14 paired samples, the tumor tissue total cholesterol content was increased after 2 weeks of atorvastatin treatment. In the remaining three cases, the tumor tissue total cholesterol content was lower than before treatment (Fig. 2). However, no statistically significant differences in the levels of total cholesterol pre- and post-treatment were observed [*P* = 0.11 (Wilcoxon signed-rank test)]. Also, the remaining 28 un-paired post-atorvastatin treatment samples from the trial were analyzed for their total cholesterol content, ranging between 4.72 and 21.86 μg total cholesterol/10 mg tissue, median value 9.49 μg (Additional Figure 1). Among all 42 post-treatment samples, the range was 4.72 to 46.35 *μ*g total cholesterol/10 mg tissue, with a median value of 11.67 *μ*g.

### Patient and tumor characteristics according to intratumoral cholesterol content

The tumor characteristics of the 42 patients who completed all study parts and the cohort of 14 patients evaluated for paired intratumoral cholesterol levels were similar (Table 1). To explore the associations between intratumoral cholesterol levels and patient and tumor characteristics, the 42 post-treatment samples were divided into tertiles of intratumoral cholesterol content: tertile 1, 4.73–7.86 (μg/10 mg tissue); tertile 2, 8.33–16.02 (μg/10 mg tissue); and tertile 3, 16.04–46.35 (μg/10 mg tissue). Tumor samples in tertile 3 were considered the cholesterol-rich group of tumors, whereas tertiles 1 and 2 served as the joint cholesterol-low group. Table 2 summarizes patient and tumor characteristics according to intratumor cholesterol levels. There were no statistically significant differences between cholesterol-rich tumors and cholesterol-low tumors according to baseline tumor grade, mitotic index, or the expression of ER, progesterone receptor, HER2, HMGCR, or LDLR.

**Table 1:** Patient and tumor characteristics

	Completed all study parts	Complete cholesterol pairs
	*n* = 42	*n* = 14
Age years (mean, range)	63 (35-89)	68 (50-83)
Tumor size mm (mean, range)	21 (6-33)	23 (13-32)
Tumor grade (NHG)		
I	9 (21%)	4 (29%)
II	17 (41%)	3 (21%)
III	16 (38%)	7 (50%)
Mitotic index		
1	23 (55%)	6 (43%)
2	5 (12%)	1 (7%)
3	14 (33%)	7 (50%)
ER (*n* = 30), baseline		
Positive	27 (90%)	14 (100%)
Negative	3 (10%)	0 (0%)
PR (*n* = 30), baseline		
Positive	24(80%)	11 (79%)
Negative	6 (20%)	3 (21%)
HER2 (*n* = 29), baseline		
0	7 (24%)	4 (32%)
1+	10 (34%)	5 (38%)
2+	7 (24%)	2 (15%)
3+	5 (17%)	2 (15%)
Ki67 index (*n* = 26), baseline		
Low	15 (58%)	7 (54%)
High	11 (42%)	6 (46%)
HMGCR (*n* = 38)		
Negative	14 (37%)	5 (38%)
Positive	24 (63%)	8 (62%)

*NHG* Nottingham histologic grade I-III (post-treatment pathological report), mitotic index according to Nottingham criteria (post-treatment pathological report), baseline tumor data (pretreatment): Ki67 high if >20%, HMGCR positive if any cytoplasmic staining*, ER* (estrogen receptor), *PR* (progesterone receptor), HER2 (human epidermal growth factor receptor 2)

**Table 2:** Patient- and tumor characteristics in relation to post-treatment tissue cholesterol

	Cholesterol-low	Cholesterol-rich	*P* value
	*n* = 28	*n* = 14	
Age years (median)	63.0	67.5	0.97
Tumor size mm (median)	22.5	20.5	0.47
Tumor grade (NHG)			0.26
1	7	2	
2	12	5	
3	9	7	
Mitotic index			0.10
1	17	5	
2	4	1	
3	7	7	
ER (*n* = 31)			0.31
Positive	21	7	
Negative	1	2	
PR (*n* = 31)			0.14
Positive	19	6	
Negative	3	3	
HER2 (*n* = 30)			0.97
0	5	2	
1+	7	3	
2+	5	3	
3+	4	1	
Ki67 index (*n* = 26)			0.02*
Low	14	1	
High	5	6	
HMGCR (*n* = 38)			0.87
Negative	10	4	
Positive	16	8	
Serum lipid levels, median (*n* = 42)			
LDL pre-treatment	3.76	3.21	0.20
HDL pre-treatment	1.60	1.41	0.54
Cholesterol pre-treatment	6.10	5.20	0.08
Apoliporotein B pre-treatment	1.07	0.91	0.11
Apolipoprotein A1 pre-treatment	1.65	1.72	0.84
LDL post-treatment	1.76	1.66	0.73
HDL post-treatment	1.58	1.51	0.56
Cholesterol post-treatment	3.70	3.45	0.47
Apolipoprotein B post-treatment	0.60	0.55	0.38
Apolipoprotein A1 post-treatment	1.58	1.51	0.45

*NHG* Nottingham histologic grade I-III (post-treatment pathological report), mitotic index according to Nottingham criteria (post-treatment pathological report), baseline tumor data (pretreatment): Ki67 high if > 20%, HMGCR positive if any cytoplasmic staining, *ER* (estrogen receptor), *PR* (progesterone receptor), *HER2* (human epidermal growth factor receptor 2) *P* values: Mann Whitney *U* test, linear-by-linear association chi-square test

In both pre- and post-atorvastatin treatment blood samples, the median level of most of the serum lipid levels were higher among patients with cholesterol-low tumors compared to the patients with cholesterol-rich tumors, however, without reaching statistical significance (Table 2).

**Fig. 3:**
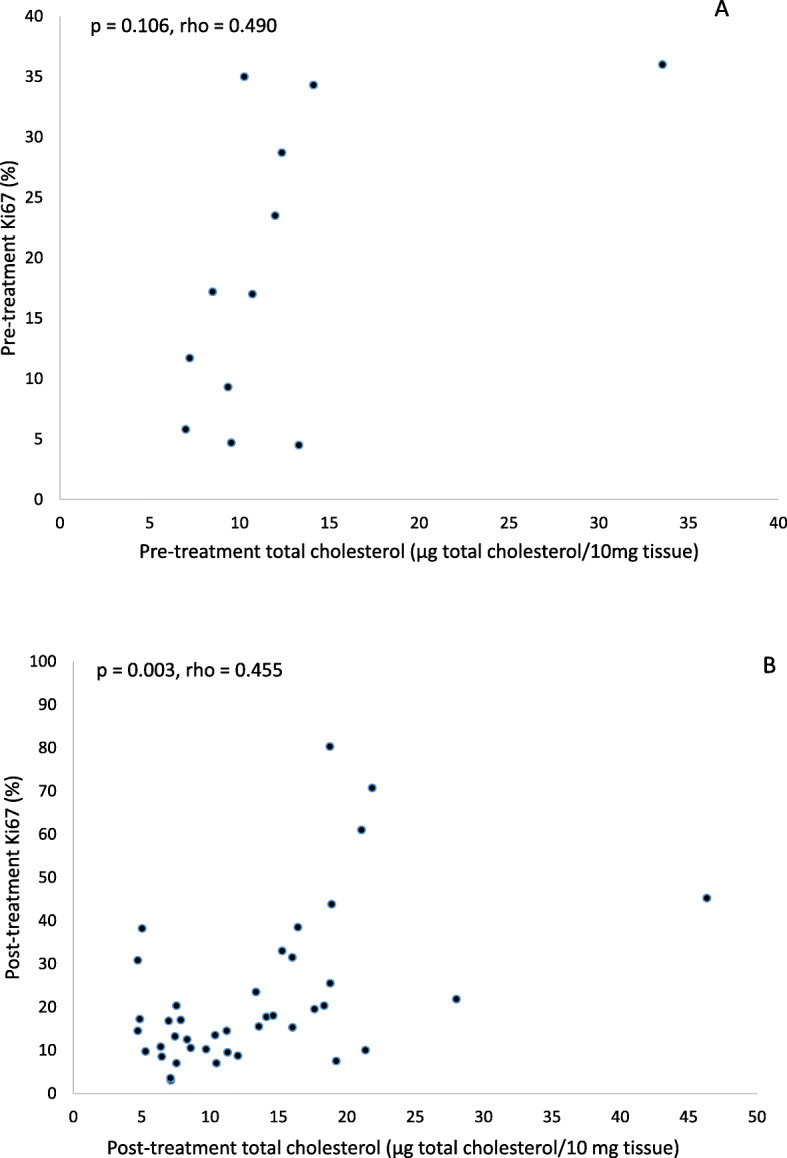
Correlation between tumor tissue total cholesterol and Ki-67. **a** Between pre-treatment tumoral total cholesterol and pre-treatment Ki-67, a non-significant positive correlation was found. **b** Between post-treatment tumoral total cholesterol and post-treatment Ki-67, a significant positive correlation was found

The baseline Ki-67 levels were higher in the cholesterol-rich tumors compared to cholesterol-low tumors which motivated further analysis of the correlation between intratumoral cholesterol levels and Ki-67. Between pre-treatment intratumoral cholesterol levels and pre-treatment expression of Ki-67, the correlation coefficient was 0.49 (Spearman’s rho), but the correlation was non-significant (*P* = 0.11, Fig. 3a). A positive association was observed between post-treatment intratumoral cholesterol levels and post-treatment Ki-67, as illustrated in Fig. 3b (*P* = 0.003, correlation coefficient 0.46 (Spearman’s rho)).

### Expression of the LDL receptor pre- and post-atorvastatin treatment

**Fig. 4:**
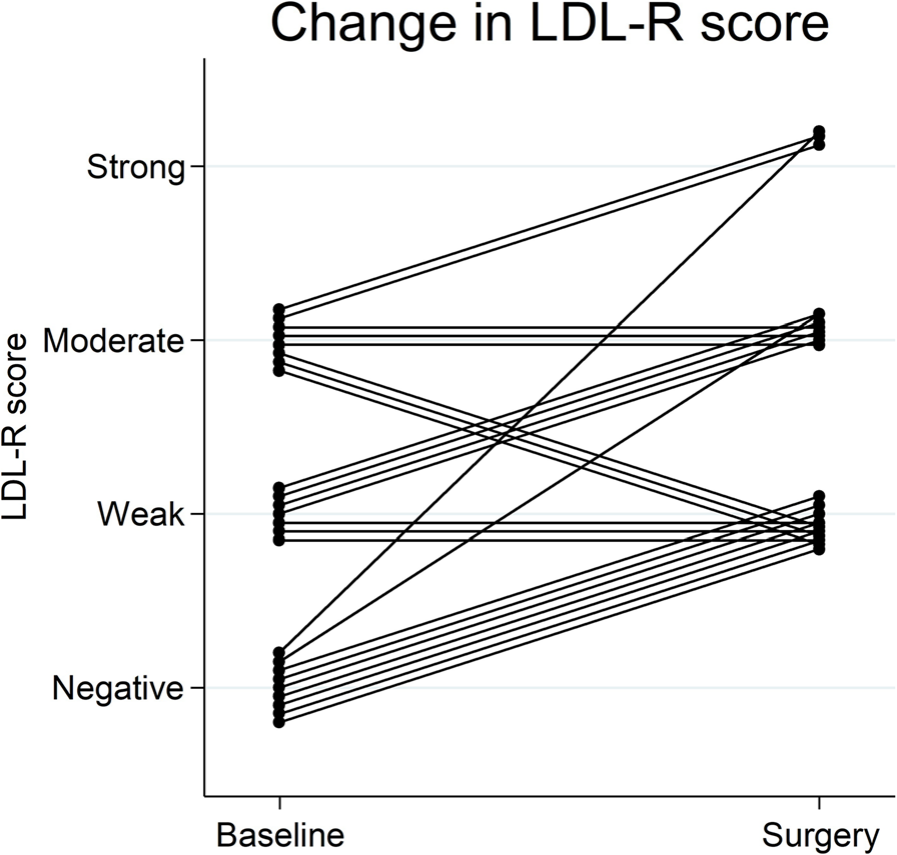
Change in tumor tissue LDLR score. Change in tumor expression of LDLR from baseline (i.e., before atorvastatin treatment) to time of surgery (i.e., after atorvastatin treatment). A significant increase in the expression of the LDLR was found (*P* = 0.004, Wilcoxon matched-pairs signed-rank test)

Although non-significant, there was a trend toward increased intratumoral cholesterol levels following atorvastatin treatment (Fig. 2). Therefore, we next investigated a possible correlation between atorvastatin treatment and expression of the LDLR protein. Immunohistochemical evaluation of LDLR was achievable in 24 paired tumor samples. Among the pre-atorvastatin samples, 41% were negative for LDLR in tumor cells. Following atorvastatin treatment, all samples stained positive for LDLR to different extents, and there was a significant increase in the expression of the LDLR compared with paired pre-treatment tumors (*P* = 0.004, Wilcoxon matched-pairs signed-rank test) (Fig. 4).

### Patient and tumor characteristics according to the LDL receptor protein expression

Table 3 summarizes patient and tumor characteristics according to baseline LDLR expression, where no statistically significant differences were found. To explore which tumors were upregulating the LDLR, the correlation between the change of the LDLR expression and Ki-67 was analyzed, and a suggestive, positive correlation between increased LDLR and post-treatment Ki-67 was found [*P* = 0.005, correlation coefficient 0.57 (Spearman’s rho)], as well as a non-significant positive correlation between the change of the LDLR and the change of Ki-67 [*P* = 0.094, correlation coefficient 0.37 (Spearman’s rho)]. However, no correlation was found between the change of the LDLR and the change of intratumoral cholesterol levels or the change of expression of HMGCR. Neither was any correlation found between the change of LDLR and the intratumoral cholesterol levels.

**Table 3:** Association of tumor characteristics and baseline LDL receptor expression

	Negative	Weak	Moderate	*P* value
	*n* = 11	*n* = 7	*n* = 9	
Age years (median)	62	67	67	0.44
Tumor size mm (median)	20	22	25	0.43
Tumor grade (NHG)				0.28
I	2	1	3	
II	4	2	3	
III	5	4	2	
Mitotic index				0.33
1	6	1	6	
2	0	2	1	
3	5	3	1	
ER (*n* = 31)				0.25
Positive	9	7	8	
Negative	2	0	0	
PR (*n* = 31)				0.27
Positive	9	5	7	
Negative	2	2	1	
HER2 (*n* = 30)				0.56
0	3	1	2	
1+	3	1	4	
2+	2	4	1	
3+	3	1	1	
Ki67 index (*n* = 26)				0.22
Low	5	3	6	
High	6	2	2	
HMGCR (*n* = 38)				0.25
Negative	7	2	2	
Positive	4	4	6	
Serum lipid levels (median)				
LDL pre-treatment	3.74	3.45	3.8	0.84
HDL pre-treatment	1.6	1.73	1.6	0.26
Cholesterol pre-treatment	6.2	5.55	6.1	0.67
Apolipoprotein B pre-treatment	1.01	0.95	1.17	0.22
Apolipoprotein A1 pre-treatment	1.78	1.66	1.58	0.06
LDL post-treatment	1.48	1.7	1.8	0.99
HDL post-treatment	1.63	1.64	1.5	0.41
Cholesterol post-treatment	4.1	3.5	3.4	0.43
Apolipoprotein B post-treatment	0.54	0.56	0.66	0.43
Apolipoprotein A1 post-treatment	1.71	1.51	1.54	0.19

*NHG* Nottingham histologic grade I-III (post-treatment pathological report), mitotic index according to Nottingham criteria (post-treatment pathological report), baseline tumor data (pretreatment): Ki67 high if > 20%, HMGCR positive if any cytoplasmic staining, *ER* (estrogen receptor), *PR* (progesterone receptor), *HER2* (human epidermal growth factor receptor 2) *P* values: linear-by-linear association chi-square test

### Effects of atorvastatin on gene expression in clinical samples

To further elucidate the adaptive changes in breast tumor tissue to atorvastatin treatment, analyses of gene expression data regarding selected genes of cholesterol homeostasis were performed. Good quality gene expression data were available for 25 pre- and post-treatment tumor pairs. Comparisons were made regarding *LDLR*, low-density lipoprotein receptor-related protein 1 (*LRP1*), low-density lipoprotein receptor-related protein (*LRP5*), scavenger receptor class B member 1 (*SRB1*), and cluster of differentiation 36 (*CD36*) encoding lipoprotein and fatty acid translocase receptor genes; ATP binding cassette transporter 1 (*ABCA1*) and ATP-binding cassette subfamily G member 1 (*ABCG1*) encoding enzymes involved in cholesterol efflux; sterol O-acyltransferase 1 (*SOAT1*) coding for the enzyme ACAT that converts free cholesterol into cholesterol esters; perilipin 2 (*PLIN2*) and perilipin 3 (*PLIN3*) encoding LD-associated proteins; sterol regulatory element-binding transcription factor 2 (*SREBF2*) encoding the transcription factor SREBP2 involved in cholesterol homeostasis; and sterol regulatory element-binding protein cleavage-activating protein (*SCAP*), an escort protein necessary for the activation of SREBP2 (Additional Table 1). No statistically significant differences in the mRNA expression between pre- and post-statin treatment were observed except for *ABCA1*, which was found to be suggestively downregulated (*P* = 0.026, Additional Table 1).

### In vitro experiments

#### Atorvastatin treated in vitro models and cellular proliferation assay

Atorvastatin decreased MCF-7 cell proliferation in a concentration-dependent manner, as illustrated in Additional Figure 2.

#### Atorvastatin treated in vitro models and cellular cholesterol content

To align the in vitro results with the clinical associations, analyses of the intracellular total cholesterol content in MCF-7 cells were performed. The cells were exposed to 10 μM atorvastatin for 24, 48, or 72 h, and compared to MCF-7 cells cultured in the absence of atorvastatin. In line with patient tumor data, no significant changes in the total cholesterol levels were found (Additional Figure 3).

#### Atorvastatin treated in vitro models and intracellular lipid droplets content

**Fig. 5:**
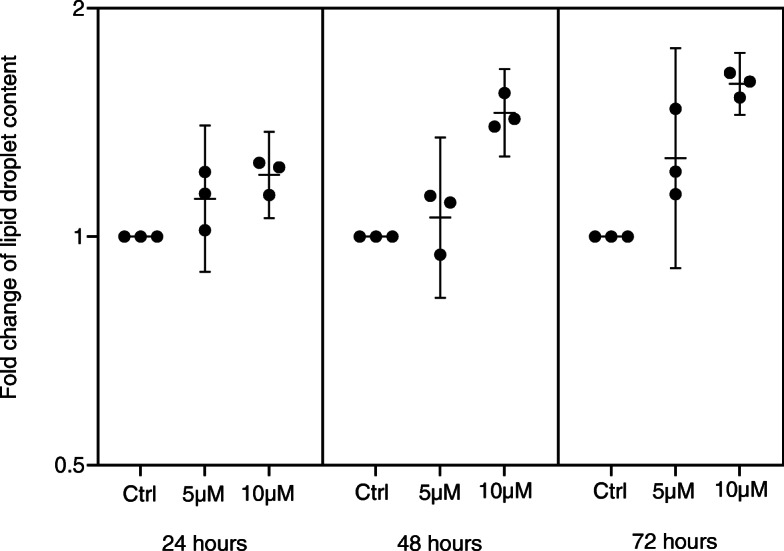
Lipid droplets in MCF-7 cells. Lipid droplets in MCF-7 cells treated with 5 or 10 μM atorvastatin for 24, 48, or 72 h, respectively, compared to untreated control. A concentration- and time-dependent increase in the abundance of intracellular LDs was observed. Values are expressed as the geometric mean ± 95% confidence interval of the geometric mean of three independent experiments

The content of LDs was analyzed in MCF-7 cells exposed to 5 or 10 μM atorvastatin for 24, 48, or 72 h, and compared to MCF-7 cells cultured in the absence of the atorvastatin. In the control cells, a sparse presence of LDs was seen, whereas in the cells exposed to atorvastatin, a concentration- and time-dependent increase in the abundance of intracellular LDs was observed (Fig. 5).

#### Atorvastatin treated in vitro models and LDLR expression

Whether LDLR expression was affected by atorvastatin at the cellular level was examined by Western immunoblotting, performed on atorvastatin-treated MCF-7 cells and compared with untreated cells. In line with the in vivo results, LDLR expression appeared higher in the atorvastatin-treated MCF-7 cells compared to controls. However, this did not reach statistical significance (Additional Figure 4).

## Discussion

In this translational breast cancer trial, we investigated the effect of short-term, high-dose atorvastatin treatment on intratumoral cholesterol homeostasis, regarding the expression of LDLR and tumor tissue cholesterol levels. The results suggest a statin-induced upregulation of LDLR, whereas cholesterol levels were not significantly altered by statin treatment. Supportive in vitro studies on MCF-7 breast cancer cells cohered with the clinical results with unchanged intracellular cholesterol levels upon statin treatment. Additionally, a positive correlation between tumor proliferation and intratumoral cholesterol levels was found in the clinical samples, as well as a correlation between tumor proliferation and upregulation of LDLR.

A century ago, the first report suggesting a link between cellular cholesterol content and cancer was published [35]. Since then, several studies have shown increased levels of cholesterol in tumors as compared to normal tissue [36–38]. Different abilities to increase intracellular cholesterol have been observed in tumor cells, including increased expression of LDLR, or deficient feedback regulation by LDL [39–45]. The role of cholesterol in tumor proliferation and aggressiveness is not completely clarified, but it has been hypothesized that intracellular cholesterol is linked to a number of mechanisms connected to the malignancy of breast cancer, including reduction of the energetically costly lipid synthesis, induction of pro-tumorigenic signaling, and increase of the membrane synthesis and rigidity [46, 47].

Statins predominantly target the hepatocytes, where they inhibit HMGCR, thus lowering their intracellular cholesterol levels, leading to the upregulation of LDLR and consequently a depletion of plasma levels of cholesterol, while keeping a steady-state of intracellular cholesterol [48]. However, previous data from different extrahepatic tissues show decreased intracellular levels of cholesterol after statin treatment [49–53] possibly due to lower expression of, or inability to upregulate, the LDLR. The demonstrated upregulation of LDLR and the preserved cholesterol levels following atorvastatin treatment in this study may indicate that breast tumor cells are responding to statins similarly to hepatocytes in terms of intracellular cholesterol homeostasis.

The intratumoral cholesterol levels were significantly correlated to Ki-67, the most widely used clinical marker of tumor proliferation, indicating that intratumoral cholesterol levels might be positively associated with worse prognosis of breast cancer patients. These results are in line with previous findings showing an association between intratumoral neutral lipids and tumor malignancy [47, 54]. Herein, the intratumoral cholesterol levels did not change significantly between pre- and post-statin treatment tissues. The interpretation of tissue cholesterol content data is limited by the few paired samples and the presumed heterogeneity of each sample regarding the proportion of tumor cells that was not possible to evaluate. Further, the comparison between core needle biopsies and surgical samples require some deliberations. It has been shown that Ki67 expression can vary between breast core biopsies and tumor samples taken at surgery [55], which might also apply to the expression of other biomarkers. Tumor heterogeneity may explain such differences along with factors influencing patients undergoing surgery; i.e., physiological stress and treatments that may alter host metabolism and finally differences in sample handling [56]. For example, the devascularization of a tumor during resection may lead to an increase in the degree of hypoxia, with metabolic consequences [56], emphasizing the importance of freezing the samples instantly at the time of biopsy. Adjacent to the malignant cells, the tumor microenvironment includes, e.g., cancer-associated fibroblasts, infiltrating immune cells, adipocytes, nerve cells, and endothelial cells [57]. Molecular communications between tumor cells and adjacent cells of the tumor microenvironment are of importance for the development, spread, and response to anti-cancer treatment [58]. Further, lipid levels depend on cellular oxygenation status and extracellular pH that vary between different tumor areas and also correlate with tumor aggressiveness [6]. Thus, evaluating the whole tumor complexity as a unit aggravates the interpretation of the direct effect of atorvastatin to the cholesterol content in tumor cells exclusively, but is not without significance. The supporting in vitro studies, where the cancer cells are analyzed exclusively, revealed no significant changes regarding the intracellular total cholesterol levels by atorvastatin, but statin treatment induced an increase in LD abundance in MCF-7 cells. These divergent results could be explained by the fact that the difference in total cholesterol was too small to be captured by the cholesterol assay, or by redistribution of cholesterol from various cellular membranes into LD stores. This would be consistent with our finding of decreased proliferation by atorvastatin treatment, and insufficient availability of lipids required for rapid proliferation, as shown previously [59, 60]. Furthermore, LDs are composed of both cholesteryl esters and triacylglycerol, and when statins inhibit the de novo synthesis of cholesterol; compensatory induction of LDL uptake via the upregulation of LDLR might result in increased storage of LDL-derived triacylglycerol. The induction of LDs can also be due to a general stress response of the cells [21]. An increased amount of LDs has been found to be correlated with increased aggressiveness of cancer [61], and some studies also show that LDs have a role in many aspects of cancer development [6, 20, 62]. Analyses of gene expression data from the clinical samples regarding selected genes related to LDs show no indication of elevated esterification of cholesterol, or elevated levels of the LD coating proteins perilipin-2 or -3 following atorvastatin treatment and do not support an increase in the LDs in the clinical samples. Attempts were made to assess LD density on cryosections of patient tumors but turned out to be technically challenging due to high background staining and heterogeneity.

Statin use has been shown to reduce the risk of recurrence and mortality of breast cancer [23, 63–66]. The exact mechanism behind this secondary preventive effect is not known, but cellular experiments have shown that statins exert pleiotropic effects through multiple mechanisms and affect breast cancer cells by increasing apoptosis, inhibiting angiogenesis, and inducing cell cycle arrest [24, 67]. If the upregulation of LDLR seen in this study contributes to the breast cancer preventative effect of statins cannot be concluded based solely on these results. As shown in Fig. 3, a positive correlation was found in the clinical samples between the change in LDLR expression and post-treatment Ki-67 (*r*, 0.567; *P* = 0.005), as well as between the change in LDLR expression and change in Ki-67 (*r*, 0.366; *P* = 0.094), showing that the statin-induced feedback upregulation of the LDLR is strongest in the most proliferative tumors, and in the tumors not responding to statins in terms of decrease in proliferation. A previous study investigated the effect of neoadjuvant chemotherapy on LDLR expression in locally advanced breast cancer using a polyclonal antibody, querying that the overexpression of LDLR is caused by elevated lipid-dependent membrane synthesis in highly proliferative cells, but the results showed no effect of chemotherapy and the subsequent reduction of mitosis on LDLR expression [68]. From an opposite perspective, these results indicate that the upregulation of LDLR might avert the anti-tumoral effects of statins and raises the question if statin treatment should be avoided in patients with the most apparent LDLR upregulation, or be combined with inhibition of LDLR, a target with emerging therapeutic options [69–71]. Also, preclinical studies have shown a difference in sensitivity to statins between different breast cancer cell lines, where ER-positive cell lines were found to be more insensitive to statins than ER negative [72, 73]. The relative insensitivity was found to be associated with increased accumulation of intracellular lipid droplets and fatty acid metabolism [73] and the upregulation of HMGCR and LDLR mRNA levels, which is thought to be mediated by a dysregulation of the feedback response via the SREBP-2/HMGCR/LDLR axis that counteracts the inhibition of the mevalonate pathway [72]. Since the vast majority of included patients in this trial had ER-positive breast cancer, in line with the MCF-7 cell line used for the in vitro experiments, it can be questioned if the upregulation of LDLR seen in this study is limited to ER-positive breast cancer, which needs to be further investigated in future trials. Previous research has suggested a link between tumor expression of LDLR and hypo-lipidemia in cancer patients [74], but this association could not be found in this study.

After oral statin administration, a reported dose range likely to be clinically achieved in the circulation is 0.1-3.9 μM [75], and the extrahepatic concentrations of most orally administered statins are unlikely to reach the doses utilized in vitro. Thus, the in vivo effect of statins might be smaller than the in vitro and should be accounted for in the interpretation of in vitro results. However, whether accumulated concentrations of statins occur in tumors is not known.

The gene expression of the *LDLR* was not significantly changed by 2-week atorvastatin treatment. The expression of LDLR is under strict regulation both at the transcriptional level, where it is tightly controlled by the negative feedback loop involving the proteins SREBP2 and SCAP, and at the posttranscriptional level, where it has been found to be modulated by proprotein convertase subtilisin/kexin type 9 (PCSK9) [76] and by several microRNAs that have recently emerged as key regulators of cholesterol metabolism, including LDLR [77, 78]. Moreover, N-myc downstream-regulated gene 1 (NDRG1) was recently found to regulate LDLR abundance and LDL uptake at the post-translational level [79]. The gene expression of *SREBP2* and *SCAP* showed no alteration following atorvastatin treatment in this study, indicating that posttranscriptional regulation might be involved in atorvastatin treatment-induced upregulation of LDLR protein levels. However, recently published in vitro data show that atorvastatin treatment upregulates the relative transcript levels of *LDLR* in several breast cancer cell lines [73], in line with the results at the protein level in this trial, and the method used within this trial might be too insensitive to capture a change of the *LDLR.* However, via the HMGCR, cancer cells can provide themselves with cholesterol by de novo synthesis, and the expression of HMGCR was, as earlier published in another sub-study within this trial [30], also upregulated after statin treatment, whereas the enzymatic activity should be inactivated by the presence of the drug according to the pharmacological actions of statins [80]. The evaluation of the gene expression of four lipid receptors; *LRP1*, *LRP5*, *SRB1*, and *CD36*, that could be compensatorily upregulated, revealed no significant changes after atorvastatin treatment, compatible with the hypothesis of LDLR as one of the main factors of the preserved intracellular cholesterol levels after atorvastatin treatment. However, the downregulation of *ABCA1* might indicate a contribution in terms of reduced cellular cholesterol efflux.

## Conclusions

In conclusion, the results from this breast cancer window-of-opportunity trial show statin-induced upregulation of LDLR in tumors with relatively high proliferation, as well as preserved intratumoral cholesterol levels, indicating that LDLR might play a role as a negative regulator in the statin-induced inhibition of breast cancer aggressiveness. The clinical results were supported by functional studies and contribute to the elucidation of the anti-tumoral effects of statins.

## Supplementary Information

**Additional file 1:** **Table 1.** Change in gene expression from baseline to time of surgery

**Additional file 2:** **Figure 1.** Post-treatment total cholesterol levels (un-paired samples). Amount of total cholesterol in tumor tissue, after two weeks of treatment with 80 mg atorvastatin daily.

**Additional file 3:** **Figure 2.** Proliferation of MCF-7 cells treated with atorvastatin. The proliferation of MCF-7 cells treated with 5, 10, 20, 50 and 100 μM atorvastatin for 72 h relative to untreated control. The MCF-7 cell proliferation decreased in a concentration-dependent manner.

**Additional file 4:** **Figure 3.** Total cholesterol levels in MCF-7 cells after treatment with atorvastatin. No statistical difference was found between the total cholesterol levels in MCF-7 cells after treatment with 10 μM atorvastatin for 24, 48 and 72 h, respectively, compared to MCF-7 cells cultured in absence of atorvastatin (2-way ANOVA). Values are expressed as the mean ± standard deviation of three independent experiments.

**Additional file 5:** **Figure 4.** LDLR expression in MCF-7 cells after atorvastatin treatment. (A) Atorvastatin moderately increased LDLR protein expression in MCF-7 cells treated with atorvastatin (10 μM) after 48 h of treatment, but no statistical difference was found (student’s T-test). Values are expressed as the geometric mean ± 95% confidence interval of the geometric mean of three independent experiments. (B) LDLR relative abundance was measured using Western blot analysis normalized to GAPDH.

## Data Availability

Please contact the corresponding author.

## Abbreviations

LDLR: Low-density lipoprotein receptor
LDL: Low-density lipoprotein receptor
HDL: High-density lipoprotein
SREBPs: Endoplasmic reticulum-bound sterol regulatory element-binding proteins
HMGCR: 3-Hydroxy-3-methylglutaryl-coenzyme A reductase
CE: Cholesterol esters
ACAT: Acyl-CoA acyltransferase
LD: Lipid droplets
LXR: Liver X receptor
LRP1: Low-density lipoprotein receptor-related protein 1
LRP5: Low-density lipoprotein receptor-related protein
SRB1: Scavenger receptor class B member 1
CD36: Cluster of differentiation 36
ABCA1: ATP binding cassette transporter 1
ABCG1: ATP-binding cassette subfamily G member 1
SOAT1: Sterol O-acyltransferase 1
PLIN2: Perilipin 2
PLIN3: Perilipin 3
SREBF2: Sterol regulatory element-binding transcription factor 2
SCAP: Sterol regulatory element-binding protein cleavage-activating protein
