# Supplementary figures and images for: The effect of statin treatment on intratumoral cholesterol levels and LDL receptor expression: a window-of-opportunity breast cancer trial
Source: Cancer Metab. 2020 Nov 23;8:25. doi: 10.1186/s40170-020-00231-8 (PMC7682108; doi:10.1186/s40170-020-00231-8)

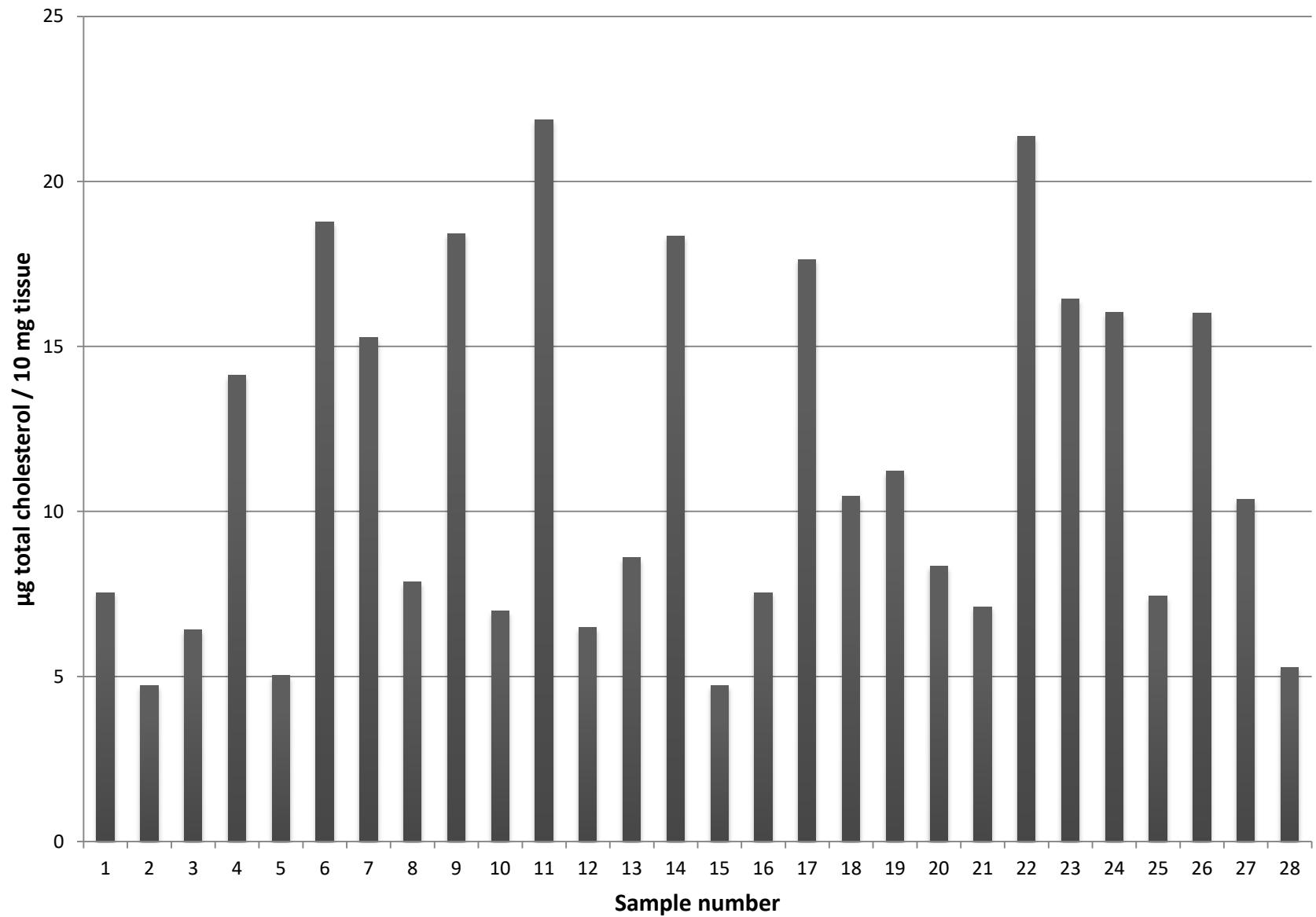

Supplement: Supplementary file 2 — Additional file 2: Figure 1. Post-treatment total cholesterol levels (un-paired samples). Amount of total cholesterol in tumor tissue, after two weeks of treatment with 80 mg atorvastatin daily. [file 40170_2020_231_MOESM2_ESM.pdf]

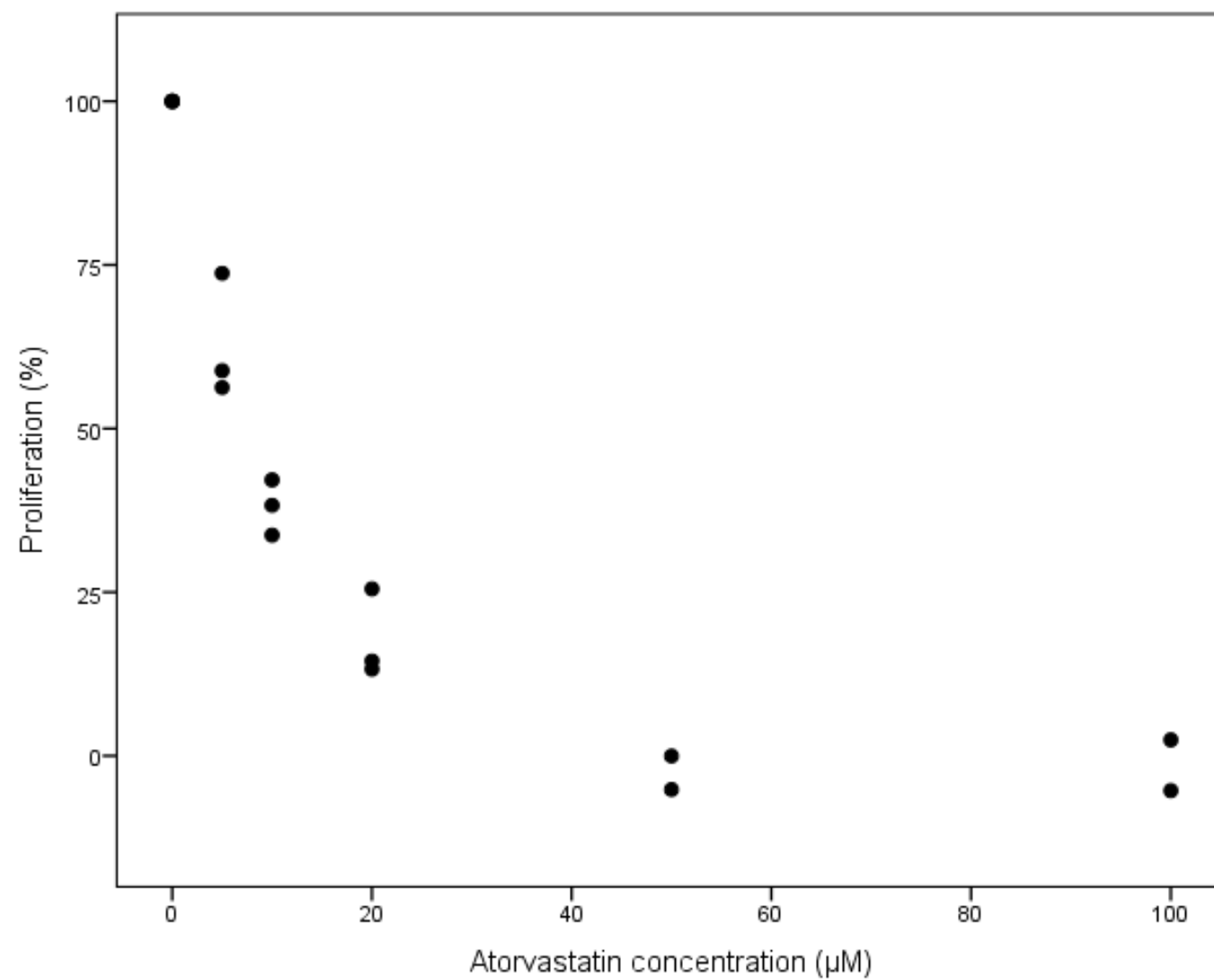

Supplement: Supplementary file 3 — Additional file 3: Figure 2. Proliferation of MCF-7 cells treated with atorvastatin. The proliferation of MCF-7 cells treated with 5, 10, 20, 50 and 100 μM atorvastatin for 72 h relative to untreated control. The MCF-7 cell proliferation decreased in a concentration-dependent manner. [file 40170_2020_231_MOESM3_ESM.pdf]

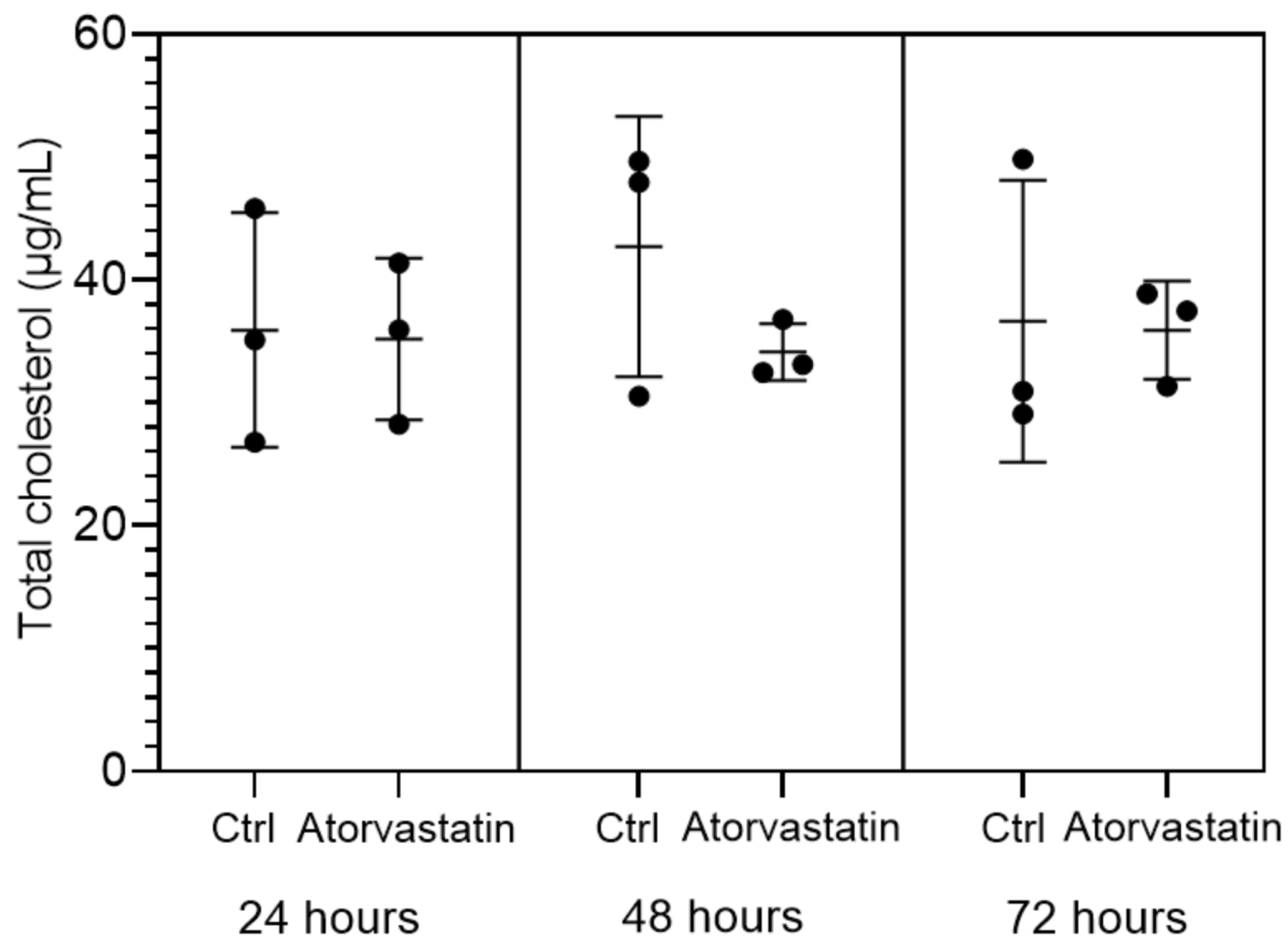

Supplement: Supplementary file 4 — Additional file 4: Figure 3. Total cholesterol levels in MCF-7 cells after treatment with atorvastatin. No statistical difference was found between the total cholesterol levels in MCF-7 cells after treatment with 10 μM atorvastatin for 24, 48 and 72 h, respectively, compared to MCF-7 cells cultured in absence of atorvastatin (2-way ANOVA). Values are expressed as the mean ± standard deviation of three independent experiments. [file 40170_2020_231_MOESM4_ESM.pdf]

A

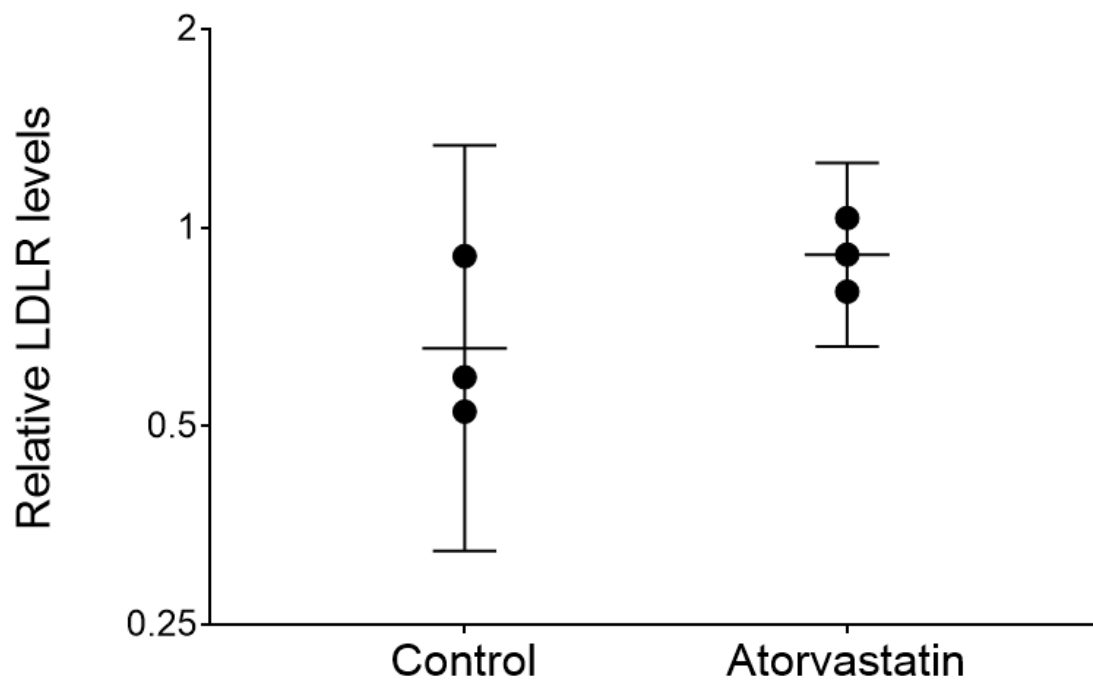

B

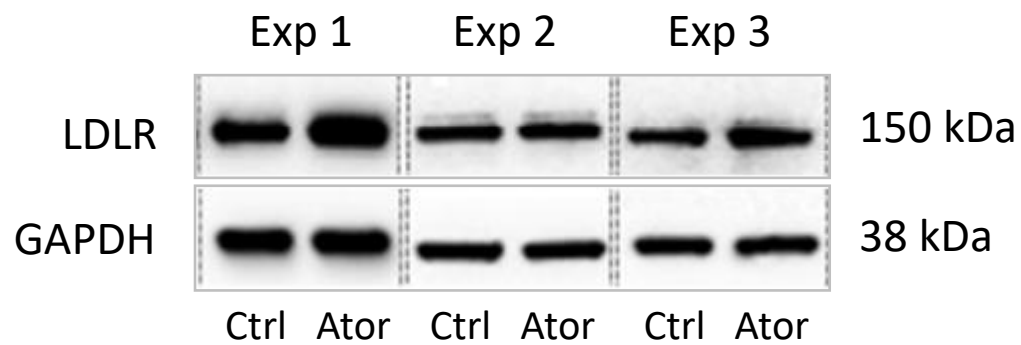

Supplement: Supplementary file 5 — Additional file 5: Figure 4. LDLR expression in MCF-7 cells after atorvastatin treatment. (A) Atorvastatin moderately increased LDLR protein expression in MCF-7 cells treated with atorvastatin (10 μM) after 48 h of treatment, but no statistical difference was found (student’s T-test). Values are expressed as the geometric mean ± 95% confidence interval of the geometric mean of three independent experiments. (B) LDLR relative abundance was measured using Western blot analysis normalized to GAPDH. [file 40170_2020_231_MOESM5_ESM.pdf]
